# 

**Published:** 1850-01-01

**Authors:** 


### THE JOURNAL

OF

### PSYCHOLOGICAL MEDICINE

AND

### MENTAL PATHOLOGY.

EDITED BY

FORBES WINSLOW, M.D.

VOL. III.

Surgeon Genl's Office LIBRARY Washington, D.C. 22047

LONDON:
JOHN CHURCHILL, PRINCES STREET, SOHO.
MDCCCL.

We must crave the kind indulgence of our numerous Correspondents this quarter. In the next Number we will make ample amends for all "sins of omission." Want of space compels us to omit our usual list of books sent for review. Correspondents wishing to have a personal interview with the Editor in town, may effect their purpose by calling at 45, Albemarle-street, Piccadilly, between the hours of one and three o'clock.

